# Supplementary material for: Anastrozole and Tamoxifen Impact on IgG Glycome Composition Dynamics in Luminal A and Luminal B Breast Cancers
Source: Antibodies (Basel). 2024 Feb 1;13(1):9. doi: 10.3390/antib13010009 (PMC10885039; doi:10.3390/antib13010009)
Supplement: Supplementary file 1 [file antibodies-13-00009-s001.zip › antibodies-2781906-supplementary.pdf]

Table S1. The table illustrates the formulas utilized to calculate derived glycan traits from integrated peaks generated by CGE-LIF and Empower 3 software.

Glycan trait	Description	Calculation
G0	agalactosylation	P14+P15+P18
G1	monogalactosylation	P16+P17+P19+P20+P21+P22+P23+P24
G2	digalactosylation	P25+P26+P27
G	galactosylation (G1+G2)	P16+P17+P19+P20+P21+P22+P23+P24+P25+P26+P27
S0	asialylation	P14+P15+P16+P17+P18+P19+P20+P21+P22+P23+P24+P25+P26+P27
S1	monosialylation	P5+P6+P7+P8+P9+P10+P11+P12+P13
S2	disialylation	P1+P2+P3+P4
S	sialylation (S1+S2)	P1+P2+P3+P4+P5+P6+P7+P8+P9+P10+P11+P12+P13
CF	fucosylation	P3+P4+P7+P8+P12+P13+P15+P18+P21+P22+P23+P24+P26+P27
CF0	afucosylation	P1+P2+P5+P6+P9+P10+P11+P14+P16+P17+P19+P20+P25
B	bisecting GlcNAc	P2+P4+P11+P13+P14+P18+P19+P20+P23+P24+P25+P27
B0	absence of bisecting GlcNAc	P1+P3+P5+P6+P7+P8+P9+P10+P12+P15+P16+P17+P21+P22+P26

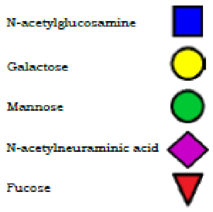

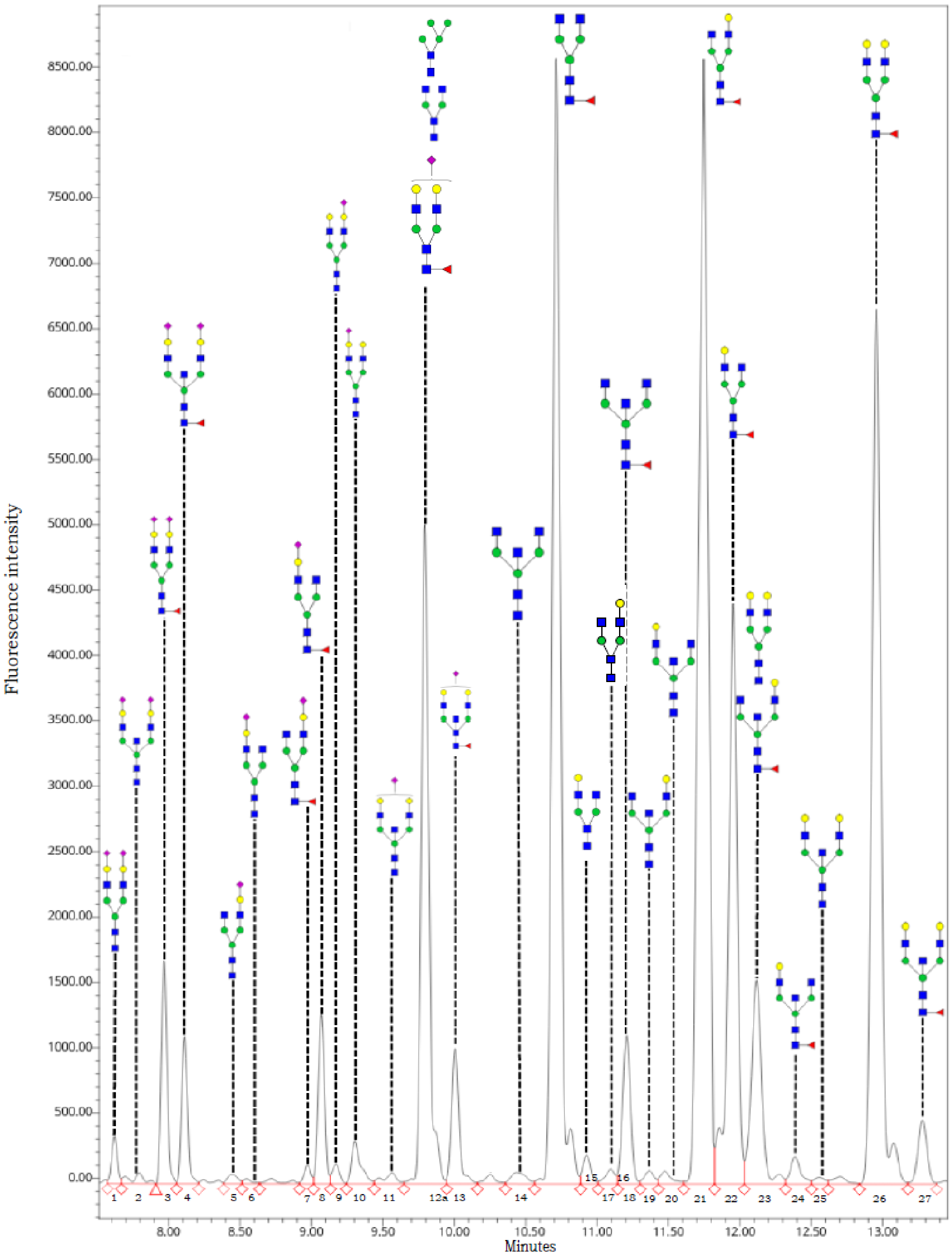

Figure S1. The image depicts the modified integrated glycan profile created using Empower 3 software. Each of the 27 peaks is clearly labelled in sequence, and the glycans associated with each peak are accurately identified.
